# Use of Olive Oil Industrial By-Product for Pasta Enrichment

**DOI:** 10.3390/antiox7040059

**Published:** 2018-04-16

**Authors:** Lucia Padalino, Isabella D’Antuono, Miriana Durante, Amalia Conte, Angela Cardinali, Vito Linsalata, Giovanni Mita, Antonio F. Logrieco, Matteo Alessandro Del Nobile

**Affiliations:** 1 Services Center of Applied Research, University of Foggia, Via Napoli 25, 71122 Foggia, Italy; lucia.padalino@unifg.it (L.P.); amalia.conte@unifg.it (A.C.); 2 Institute of Sciences of Food Production-CNR, Via G. Amendola 122/O, 70126 Bari, Italy; isabella.dantuono@ispa.cnr.it (I.D.); angela.cardinali@ispa.cnr.it (A.C.); vito.linsalata@ispa.cnr.it (V.L.); antonio.logrieco@ispa.cnr.it (A.F.L.); 3 Institute of Sciences of Food Production-CNR, Via Monteroni, 73100 Lecce, Italy; miriana.durante@ispa.cnr.it (M.D.); giovanni.mita@ispa.cnr.it (G.M.)

**Keywords:** fortified pasta, by-product, flavonoids and polyphenols, pasta quality

## Abstract

Background: During recent years food industries generally produce a large volume of wastes both solid and liquid, representing a disposal and potential environmental pollution problem. Objective: The goal of the study was to optimize, from both sensory and nutritional points of view, the formulation of durum wheat spaghetti enriched with an olive oil industrial by-product, indicated as olive paste. Methods: Three consecutive steps were carried out. In the first one, the olive paste was air-dried at low temperature, milled to record olive paste flour and properly analyzed for its biochemical composition. In the second step, the olive paste flour was added to the pasta dough at 10% and 15% (*w*/*w*). In the last step, different concentrations of transglutaminase were added to enriched pasta (10% olive paste) to further improve the quality. Sensory properties and nutritional content of enriched and control pasta were properly measured. Results: Spaghetti with 10% olive paste flour and 0.6% transglutaminase were considered acceptable to the sensory panel test. Nutritional analyses showed that addition of 10% olive paste flour to pasta considerably increased content of flavonoids and total polyphenols. Conclusions: The proper addition of olive paste flour and transglutaminase for pasta enrichment could represent a starting point to valorize olive oil industrial by-products and produce new healthy food products.

## 1. Introduction

In recent years the interest in the recovery, recycling and upgrading residues from plant food processing has increased drastically [1]. Food industries generally produce large volume of wastes both solid and liquid, that represent disposal and potential environmental pollution problems. Nevertheless, industrial by-products are also promising sources of compounds that can be recovered and re-used as valuable substances. The olive fruits in particular are found to be a good source of phenolic compounds that generally contribute to the protective effect of the olive oil. During olive oil processing, most of these phenolic compounds remain in the olive oil by-products (olive paste and olive pomace) and in the olive mill waste-water (OMWW), that are very rich in phenolic compounds widely characterized [2] for their functional properties [3]. In addition, exploitable amounts of triterpenic acids, such as oleanolic and maslinic acids have been found, mainly concentrated in the skin of fruits [4]. Therefore, the recovery of these wastes as raw materials is receiving increased attention for potential food fortification, [5] even though real applications are still very limited.

Studies have demonstrated that cereal products, for their high daily consumption are excellent carriers of bioactive substances [6]. In particular, pasta represents the best example because it is one of the most well-known products all over the world. Generally, the main pasta ingredient is durum wheat semolina that, compared to other flours, confers excellent rheological properties to the dough, superior color, good appearance and cooking quality to final pasta [7]. New formulations of pasta using non-durum wheat ingredients such as inulin, guar gum, pea fiber, locust bean gum, xanthan gum, bamboo fiber, β-glucans, bran and protein concentrates are also becoming very popular [8]. In this context, the addition of olive oil by-products to pasta could help move the oil market placement in a more competitive position and, consequently, reduce the environmental impact.

It is worth noting that the potential incorporation of by-products into food formulation could alter the sensory properties, thus suggesting careful selection of ingredients and proper technological options to be adopted [9]. Among the compounds to improve textural properties, enzymes play a major role. Transglutaminase catalyzes an acyl-transfer reaction between the g-carboxyamide group of peptide-bound glutamine residues (acyl donors) and a variety of primary amines (acyl acceptors) [10]. The covalent bond of 3ε-(g-Glu)-Lys is formed when the 3-amino group of lysine residues acts as acyl acceptor and crosslinks with other proteins. The formation of homologous and heterologous polymers among different proteins (e.g., whey, soybean, rice, casein, avenin, etc.) results from the addition of transglutaminase. It is widely utilized in the production of noodles and pasta in Japan [11].

The objective of the present study was to develop durum wheat semolina spaghetti enriched with olive paste powder, rich in bioactive molecules, such as carotenoids, triterpenic acids and polyphenols, able to exert a beneficial effects on consumer’s health. To this aim, the study was organized in three consecutive steps. In the first step the olive paste was processed to flour by drying at room temperature and subsequent milling process. In the second one, paste olive flour was added to the dough at two different concentrations (10% and 15%). The final experimental step aimed to improve the sensory quality of spaghetti enriched with olive paste flour 10% by means of transglutaminase.

## 2. Material and Methods

### 2.1. Raw Materials

Commercial durum wheat semolina was purchased from “Agostini Mill” (Montefiore dell’Aso, Italy). The olive paste was obtained by a local olive mill (Murrone, Caprarica, Lecce, Italy) from the cultivar Cellina di Nardò, milled by a Pieralisi Leopard system with DMF (Multi Phases Decanter) technology, Pieralisi Jesi (Ancona, Italy). Leopard is the two-phase decanter that can combine modern extraction technology without the addition of water. It produces a dehydrated husk similar to the one coming from a three-phase decanter, and also recovers a certain quantity of husk (olive pasta, OP) made up of wet pulp without any traces of kernel.

### 2.2. Chemicals

Tocopherol isoforms (α-, β-, and γ-tocopherol), triterpenic acids (maslinic and oleanoic acids), methyl tricosanoate, myristic, palmitic, pentadecanoic, stearic, arachidic, palmitoleic, heptadecanoic, oleic, linoleic and linolenic acids used as standard as well as all High Performance Liquid Chromatography (HPLC) grade solvents were all purchased from Sigma–Aldrich (Milan, Italy). Tocotrienol isoforms (α-, β-, and γ-tocotrienols) and carotenoid standards were purchased from Cayman chemicals (Ann Arbor, MI, USA) and CaroteNature (Lupsingen, Liestal, Switzerland), respectively. Phenolic standards used for the High Performance Liquid Chromatography-Diode Array Detector (HPLC-DAD) analysis were purchased from PhytoLab GmbH & Co. KG Dutendorfer Straße 5-7 91487 (Vestenbergsgreuth, Germany) and listed as follows: tyrosol, caffeic acid, vanillic acid, coumaric acid, vitexin, ferulic acid, oleuropein, quercetin, luteolin, apigenin.

### 2.3. Olive Paste Flour Preparation

The olive paste was dried in a dryer (SG600; Namad, Upper Marlboro, MD, USA) at 35 °C for 48 h to reach a moisture content ≤8%. After dehydration, olive paste was milled by a hammer mill (16/BV-Beccaria s.r.l., Cuneo, Italy) to obtain the olive paste flour (OPF) (≤500 μm).

### 2.4. Spaghetti Preparation

Spaghetti were produced with durum wheat semolina as described by Padalino et al. [12]. The OPF was added to the dough at 10% and 15% (*w*/*w*). In the subsequent trial, to the formulation with 10% OPF, 0.3 and 0.6% Transglutaminase Activa WM (TG) (Perrins Chemical, Triggiano, BARI, Italy) were also added, respectively. In order to ensure the solubility of the enzyme as powder, it was previously dissolved in water. Spaghetti without any addition were also manufactured and used as the reference sample (CTRL). In both steps, dough was extruded with a 60VR extruder (Namad) and dried in a dryer (SG600; Namad), as described by Padalino et al. [12]. Spaghetti samples of each batch were produced twice.

### 2.5. Sensory Analysis

Dry spaghetti samples were examined by a panel of 15 trained tasters (seven men and eight women, aged between 28 and 45 years) in order to evaluate the sensory attributes. The panelists had at least several years of experience in evaluation of pasta prior to this study; however, they were retrained for this study in a session of 2 h to be experienced in the products and terminology [13]. Appropriate descriptive terms for sensory evaluation were decided during the retraining sessions. After retraining, experienced graders were able to evaluate color and resistance to break of uncooked spaghetti and elasticity, firmness, bulkiness, adhesiveness, color, odor and taste on cooked spaghetti. The elasticity is the measure of the degree of extension of the spaghetti before the break and it is evaluated on the single sample practicing a slight traction in two points distant 10 cm. The firmness is the resistance of cooked pasta to compression by the teeth, it is measured by compressing the spaghetti strand against the palate with the tongue. The bulkiness is the measure of the degree of jamming among the spaghetti strands and it is evaluated by placing two spaghetti strands together and determining the force required for detachment. The adhesiveness is related to the formation of a surface coating made of amylose and it is evaluated by placing the spaghetti in the mouth, pressing it against the palate and determining the force required to remove it with the tongue. For the evaluation, a nine-point scale was adopted, where 1 and 9 represented the lowest and the highest intensity of a particular attribute, respectively. On the basis of the above-mentioned attributes, the panel evaluated overall acceptability of each pasta sample using a nine-point scale where 1 = dislike extremely, and 9 = like extremely. Pasta products with an overall acceptability mean score above 5 were considered as acceptable [12]. Sensory evaluation was repeated twice on two different batches of samples.

### 2.6. Extraction and Analysis of Triterpenic Acids

Triterpenic acids extraction was carried out according to Romero et al. [14]. Briefly, one gram of each sample was extracted six times using 4 mL of methanol/ethanol (1:1, *v*/*v*), the extracts were combined and re-dissolved in 1 mL of methanol and filtered through a 0.45 μm syringe filter (Millipore Corporation, Billerica, MA, USA). Quali-quantitative analysis of triterpenic acids was carried out by the method of Lozano-Mena et al. [15] slightly modified, using an Agilent 1100 Series HPLC system equipped with Phenomenex-luna 5 μm C18 (2) 100 Å column (250 × 4.6 mm). To record HPLC runs the Agilent ChemStation software was used. The mobile phases were: acetonitrile (A) and 1% (*v*/*v*) H_3_PO_4_ in water (B). The gradient elution was as follows: 0 min, 60% A and 40% B; 0–5 min, 50% A and 50% B; 5–10 min, 40% A and 60% B; 10–20 min, 30% A and 70% B; 20–25 min, 25% A and 75% B; 25–30 min, 20% A and 80% B; 30–35 min, 80% A and 20% B, and 40 min, 0% A and 100% B. The flow rate was 1.0 mL/min and the column temperature was maintained at 30 °C. Absorbance was registered at wavelengths of 210 nm. Triterpenic acids were identified and quantified by the retention time, spectra and response factors of the pure standards.

### 2.7. Tocochromanols and Carotenoids Extraction and Analysis

Tocochromanols and carotenoids were extracted by mild saponification, as described by Panfili et al. [16] slightly modified. Two grams of sample were saponified with 2 mL methanolic KOH (60%, *w*/*v*), 2 mL ethanol (20%, *v*/*v*), 1 mL NaCl (0.1% *w*/*v*) and 5 mL BHT (0.05%, *w*/*v*) in acetone. After a digestion time of 30 min at 60 °C the samples were cooled and 15 mL sodium chloride solution (1%, *w*/*v*) was added. The mixture was then extracted twice with 15 mL *n*-hexane/ethyl acetate (9/1, *v*/*v*). The upper layer was dried under nitrogen flux and was re-dissolved in 1 mL ethyl acetate, filtered through a 0.45 μm syringe filter. Quali-quantitative analyses of tocochromanols and carotenoids were carried out according to Fraser et al., [17] with some modifications, using an Agilent 1100 Series HPLC system equipped with a reverse-phase C30 column (5 μm, 250 Å–4.6 mm) (YMC Inc. Wilmington, NC, USA) at 25 °C. The mobile phases were: methanol (A), 0.2% ammonium acetate aqueous solution/methanol (20/80 *v*/*v*) (B), and tert-methyl butyl ether (C). The gradient profile was as follows: 0 min, 95% A and 5% B; 0–12 min, 80% A, 5% B, and 15% C; 12–42 min, 30% A, 5% B, and 65% C; 42–60 min, 30% A, 5% B, and 65% C; 60–62 min, 95% A, and 5% B. The flow rate was 1.0 mL/min and the column temperature was maintained at 25 °C. Absorbance was registered by DAD at wavelengths of 290 nm and 475 nm for tocochromanols and carotenoids, respectively. Tocochromanols and carotenoids were identified and quantified by the retention time, spectra and response factors of the pure standards.

### 2.8. Extraction and Fatty Acids Analysis

The lipids were extracted from olive paste and pasta using acid hydrolysis [18]. A volume of 2.5 mL HCl (25%, *v*/*v*) was added to 0.5 g of each sample. The mixture was incubated at 80 °C for 30 min in a water bath and then rapidly cooled in an ice bath before the addition of 1.5 mL diethyl ether and centrifuged for 7 min at 4000 rpm. The extraction procedure was repeated twice and the organic layers were collected. One mL of extract was supplemented with 75 µL of methyl tricosanoate (1 mg/mL) as internal standard and dried under nitrogen flux. The residues were re-dissolved in 250 µL CHCl_3_ and 250 µL acetyl-chloride in methanol (3% *v*/*v*) (Instant Methanolic HCl kit-Alltech, Deerfield, Illinois, USA) were added. The samples were heated at 60 °C in a water bath for 30 min. The fatty acid methyl esters (FAMEs) were dried under nitrogen at 40 °C and the residues re-dissolved in 500 µL of hexane and analyzed by GC-MS. The analysis of fatty acids was performed on an Agilent 5977E GC/MS system (Boblingen, Germany) as described by Durante et al. [19].

### 2.9. Extraction and Analysis of Free and Total Phenolic Compounds

The phenolic compounds were extracted from semolina, olive paste flour (OPF), spaghetti CTRL and spaghetti enriched with 10% OPF in two separated fractions: soluble free and bound, following the method reported by Mattilla [20] without acid hydrolysis. The difference between total and free polyphenols represented the bound phenolic compounds. HPLC-DAD analysis was performed using the Agilent 1260 infinity system, equipped with a 1260 binary pump, 1260 HiP Degasser, 1260 TCC Thermostat, 1260 Diode Array Detector and Agilent Open Lab Chem Station Rev C.01.05 (35) software. The UV–visible absorption chromatogram was detected at 280 nm, 325 nm and 360 nm. The separation was performed by gradient elution on a 4.6 × 250 mm reversed phase Luna C-18 (5 μm) column (Phenomenex Torrance, California, USA). The elution was performed using methanol (eluent A) and water/acetic acid 95:5 (eluent B). The gradient profile was: 85–60% B (0–25 min), 60% B (25–30 min), 60–37% B (30–45 min), 37% B (45–47 min), 37–0% B (47–52 min). The flow rate was 1 mL/min and the injection volume was 25 μL. Phenolics were identified and quantified by the retention time, spectra and response factors of the pure standards.

### 2.10. Pasta Characteristics Determination

The optimal cooking time (OCT) of pasta and the cooking loss (the amount of solid substance lost into the cooking water), were both evaluated according to the American Association for Clinical Chemistry (AACC) approved method [21]. The swelling index and the water absorption of cooked pasta (grams of water per gram of dry pasta) were determined according to the procedure described by Padalino [12]. Hardness and adhesiveness were determined by a Texture Analyzer (Zwick Roell Group, Ulm-Germany; model Z010) equipped with a stainless steel cylinder probe (2 cm diameter). The hardness (mean maximum force, N) and the adhesiveness (mean negative area, Nmm) of cooked spaghetti were measured according to the procedure described by Padalino [12]. Six measurements for each sample were performed.

### 2.11. Statistical Analysis

Analytical results were reported as the mean value ± standard deviation of three independent replicate experiments (*n* = 3). Statistical analysis was based on a one-way ANOVA test. Tukey’s post hoc method was applied to establish significant differences between means (*p* < 0.05). All statistical comparisons were performed using SigmaStat version 11.0 software (Systat software Inc., Chicago, IL, USA). Experimental data on pasta characteristics were compared by one-way analysis of variance (ANOVA). A Duncan’s multiple range test, with the option of homogeneous groups (*p* < 0.05), was carried out to determine significant differences between samples. STATISTICA 7.1 for Windows (StatSoft, Inc, Tulsa, OK, USA) was used.

## 3. Results and Discussion

The work was organized in three consecutive steps: first, the OPF flour was produced and characterized; afterwards, it was added to durum wheat semolina dough in different concentrations; finally, transglutaminase was used to improve the sensory quality of OPF enriched pasta. In the following paragraphs, the above-mentioned steps will be discussed separately.

### 3.1. Step 1: Biochemical Composition of Semolina and OPF

As reported in Section 2, the OPF was prepared by first dehydrating the olive paste at low temperature to reduce the risk of losing thermo-unstable compounds. Afterwards, dried olive paste was milled to four. The biochemical composition of both OPF and durum wheat semolina are reported in Table 1. OPF and durum semolina showed similar total carotenoids (5.36 and 5.66 µg/g Dry Weight (DW), respectively) with lutein as the most abundant one. The quali-quantitative characterization of tocochromanols evidenced a remarkable difference between samples. Olive paste was characterized by the exclusive presence of α-tocopherol (107.17 µg/g DW), the most biologically active form of vitamin E. On the contrary, semolina showed the highest β-tocotrienol content (11.95 µg/g DW), in agreement with data reported by Laddomada [22]. Olive fruits are rich in triterpenic acids, such as oleanolic and maslinic acids, present in the epicarp, in the endocarp, in the wood shell and in the seeds of olives [4]. Total triterpenic acids represented one percent weight of olive paste flour analyzed, the concentration of maslinic acid (6.76 mg/g DW) was higher than the oleanolic acid (3.65 mg/g DW). Table 1 also reports the fatty acid profile (expressed as relative percentage) of OPF and semolina. In the OPF, oleic acid was the most abundant fatty acid, contributing for 57.20% to the total, followed by palmitic (21.40%) and linoleic (12.50%) acids. In the semolina, palmitic (43.93%) and linoleic acids (35.20%) were the main saturated and unsaturated fatty acids, respectively, in agreement with Belleggia [23].

**Figure 1:**
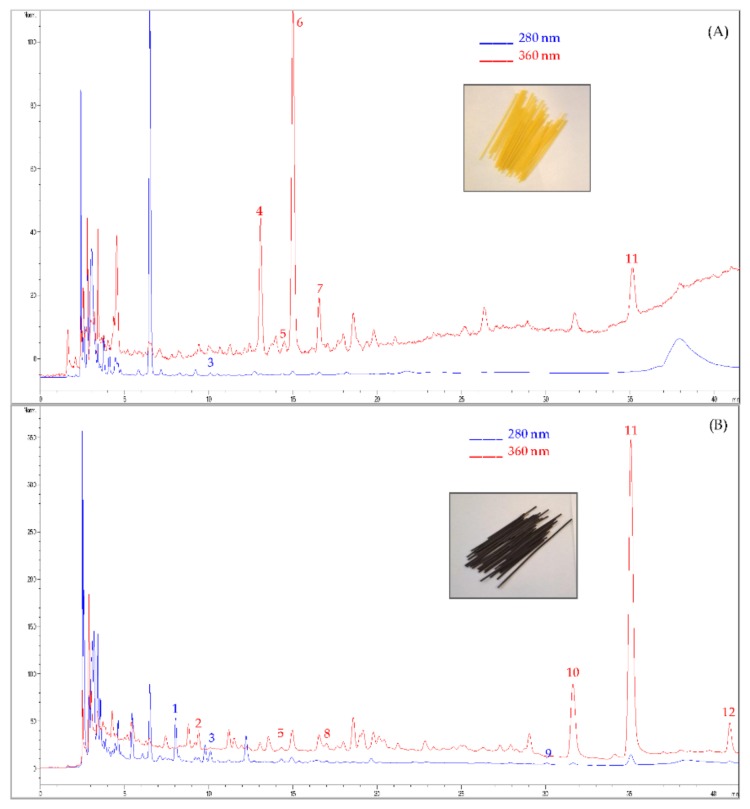
High Performance Liquid Chromatography-Diode Array Detector (HPLC-DAD) profile of control spaghetti (**A**) and spaghetti enriched with 10% olive paste flour (**B**). Polyphenols identification: 1. tyrosol, 2. caffeic acid, 3. vanillic acid, 4. apigenin derivative, 5. coumaric acid, 6. vitexin, 7. ferulic acid, 8. caffeic acic derivative, 9. oleuropein, 10. quercetin, 11. luteolin, 12. apigenin.

The polyphenols composition of OPF and semolina is also shown in Table 1. Phenolic acids and flavonoids are present in plant in both free and conjugated/bound forms; while the free polyphenols are extracted using a hydro-alcoholic solution at room temperature, for conjugated and bound compounds an alkaline or acidic hydrolysis treatment is necessary. The OPF resulted very rich in free phenolic compounds identified by HPLC-DAD analysis (Figure 1), with a total amount of 2616 μg/g DW. It is interesting to note the presence of several flavonoids; among them, luteolin and quercetin aglycons were the most abundant (533.59 and 308.23 μg/g DW, respectively). Among the phenolic acids, caffeic, vanillic and coumaric acids were detected; these compounds although present at very low concentrations, are noteworthy for their biological activity [24]. In addition, tyrosol, characteristic compound of olive products, was the most abundant phenols recovered in the OPF (936.12 μg/g DW), followed by oleuropein (371.42 μg/g DW). The presence of the latter could be related to the early ripeness degree of olives, as demonstrated by the absence of hydroxytyrosol, hydrolysis product of oleuropein, normally recovered in the ripe olives [25]. The fraction of OPF not solubilized by the aqueous organic solvent (non-extractable polyphenols) showed that caffeic acid was the major compound produced by alkaline hydrolysis (576.70 μg/g DW) followed by coumaric and ferulic acids. The presence of low molecular weight phenolics after alkalin treatment are in agreement with results also obtained by Arranz [26], using different plant-derived foods, that demonstrated the bonds break between arabinoxylan and ferulic acid in cell wall plants, with the release of ferulic acid. Further, the total amount of phenol compounds (extractable and non-extractable) recovered was 3249.30 μg/g DW, underlining the high composition of bioactive compounds in the OPF.

The semolina sample used in this work was also analyzed for the phenolic composition (Table 1). The main amount of polyphenols was recovered as bound form, with the ferulic acid as the most abundant (72.50 μg/g DW), followed by sinapic, coumaric, 4-hydroxybenzoic, syringic, vanillic acids and caffeic derivatives acid. In addition, in the soluble free fraction some flavonoids, such as apigenin derivative, vitexin, luteolin and apigenin, were recognized.

### 3.2. Step 2: Sensory Quality of Spaghetti Enriched with OPF

The sensory properties of dry spaghetti investigated in this work are shown in Table 2. Data highlight that the overall quality of both uncooked and cooked control samples (CTRL) was higher than pasta enriched with OPF. In fact, the overall quality of these samples decreased as the olive paste flour increased. In particular, poor color (dark green) and break to resistance were found in the uncooked spaghetti with 15% OPF. Pasta color is an important parameter for the assessment of pasta quality, and generally, consumers prefer pasta with a bright yellow color [27]. Regarding the cooked samples, the pasta with 15% OPF recorded low elasticity and firmness, thus receiving a global score for overall quality slightly under the acceptability threshold (4.42). Most probably, this is due to the inclusion of fibers from olive paste that promoted the formation of discontinuities or cracks in the pasta strand, thus weakening its structure [28]. A weak or discontinuous protein matrix results in a protein network that is too loose and permits greater amount of leaching during starch granule gelatinization, causing increased adhesiveness and bulkiness [12]. In fact, the sample enriched with 15% OPF recorded the smallest adhesiveness and bulkiness values. In addition, spaghetti enriched with 15% OPF had very intense taste and odor that further compromized their quality. Based on the sensory evaluation, spaghetti sample enriched with 10% OPF was selected for the subsequent optimization.

### 3.3. Step 3: Effect of Transglutaminase Addition on Spaghetti Enriched with OPF

#### 3.3.1. Sensory Quality

Sensory properties of dry spaghetti with and without TG are listed in Table 3. Results highlighted that the addition of TG significantly improved the sensory quality of pasta. In particular, Table 3 shows that the overall quality of 10% OPF with 0.6% TG sample recorded an overall quality score very similar to the CTRL sample, for both uncooked and cooked spaghetti. As reported in the Table 3, the overall quality rose with the increase of TG amount, above all for elasticity, firmness, adhesiveness and bulkiness. These results are in agreement with Kang [29], who also found that TG addition to noodles prevented texture deterioration after cooking, increased hardness and elasticity and decreased stickiness. It is expected that the cross-linking introduced by TG are heat-stable and reduced leaching of starchy materials [30]. Regarding the color, odor and taste, no significant differences were found among samples.

The cooking performances in terms of optimum cooking time, cooking loss, water absorption, swelling index, hardness and adhesiveness are given in Table 4. The OCT value of the spaghetti with OPF was smaller than the other samples. It is conceivable that a physical disruption of the gluten matrix due to the presence of fibers may have facilitated the penetration of water into the pasta core [12]. The cooking loss results suggest that the incorporation of OPF prevented the formation of the gluten network and therefore, negatively influenced pasta cooking quality. In fact, while the 10% OPF sample recorded the highest cooking loss, the supplementation of spaghetti with TG resulted in a significant decrease of cooking loss, indicating improved spaghetti quality, above all when 0.6% TG was used. Literature data also demonstrated that cooking loss was reduced by TG treatment of noodle dough because starch is better held in the gluten network and reduced solid loss into the boiling water [11]. The significant decrease in cooking loss of the TG-supplemented spaghetti can be also explained in terms of formation of covalent cross-links catalyzed by TG that reduced the solids released during cooking. Cross-linked proteins might form a network around the starch granules and encapsulate them during cooking and restrict the diffusion of starch. Concerning the swelling index and the water absorption, results showed that the 10% OPF sample showed the highest values. Increase of water absorption and swelling index with OPF addition could be explained by the high fiber content addition. Lončarić [28] also observed a rise in water absorption for pasta enriched with apple flour with respect to control pasta. The addition of TG caused a decline of these two parameters. Most probably, the structural change of gluten, due to the TG cross-linkage, increased water-holding capacity and TG had a profound influence on the decrease of water absorption [31]. Wu and Corke [30] also speculated that the action of TG leads to an increased water-holding capacity due to the increased hydrophilicity of gluten proteins by the hydrolysis of glutamine residues to glutamic acid. As result, the strong protein network prevents water diffusion into the starch granules, thus limiting the swelling index [32]. In fact, the 10% OPF sample with 0.6% TG had the lowest swelling index compared to the other samples. No differences among samples were recorded in terms of adhesiveness. From the Table 4 it can be also inferred that spaghetti loaded with TG showed higher hardness than 10% OPF sample, due to the stronger and tighter protein network, also responsible for limiting the excessive water uptake during cooking [33]. The decrease in surface stickiness of spaghetti samples can be attributed to the protein network created through the cross-linking of gluten proteins catalyzed by the TG that might be responsible for preventing leaching of starchy material to the surface of spaghetti strands, thus also decreasing the stickiness. Kuraishi [11] also reported that starch granules in semolina dough are better held within the gluten network that is strengthened by the addition of TG and therefore, it would be responsible for the surface less sticky of noodles and for the reduction in bulkiness. These data are in agreement with sensory analysis because the panel test also assessed that OPF spaghetti were less adhesive compared to the sample without TG.

#### 3.3.2. Biochemical Composition

Considering that the OPF spaghetti sample had a good sensory quality score, its biochemical composition, compared to the CTRL, was evaluated (Table 5). Results showed an enrichment of α-tocopherol, α- and β-carotene, maslinic and oleanolic acids in OPF spaghetti, respect to the CTRL. The ratio of polyunsaturated (PUFA) to saturated fatty acids (SFA) is commonly used to assess the nutritional quality of the lipid fraction of foods [19] and according to the current dietary guidance for healthy nutrition, PUFA/SFA ratio above 0.4–0.5 is considered optimal [34]. In OPF spaghetti the PUFA/SFA ratio resulted higher (1.16) than the CTRL (0.69). The total polyphenols content in dry spaghetti samples increased from 82.39 μg/g DW to 245.08 μg/g DW after 10% OPF enrichment (Table 5). Generally, the majority of studies on functional pasta were related to the whole-wheat flours or adding flours with different cereals typologies (barley, oat, rice) [35]. Instead, very few works are focused on pasta enriched by polyphenols. Sun-Waterhouse [36] showed that the addition of elderberry juice in fibre-enriched pasta increased the total extractable polyphenols contents about 60 times. In our study, the amount of total free phenolics in the enriched samples, was almost 50 times higher respect to the CTRL (107.61 and 2.3 μg/g DW, respectively), with tyrosol and oleuropein as the most abundant. In addition, it is interesting to underline that the spaghetti preparation process preserved an aliquot of flavonoids, such as apigenin, luteolin and quercetin present in the OPF, 15 times higher than CTRL. Further, the OPF spaghetti showed the highest amount of phenolic acids, mainly present as conjugated and bound forms, followed by the free form. These results are in agreement with the study of Verardo [35] on spaghetti enriched with barley coarse fraction. These authors demonstrated that the functional spaghetti had high fiber content and at the same time a high antioxidant activity for the presence of flavan-3-ols and phenolic acids.

## 4. Conclusions

In this study, the effects of addition of an olive oil industrial by-product on chemical composition, cooking and sensory quality of durum wheat semolina pasta were evaluated. The best amount to enrich pasta with olive paste corresponds to 10% (*w*/*w*) but further technological options needed to be applied to make the product more acceptable. Therefore, 0.6% TG addition increased the overall quality of pasta in terms of elasticity, firmness, adhesiveness and bulkiness. Additionally, the supplementation of spaghetti with 0.6% TG resulted in a significant decrease of cooking loss, swelling index and water absorption. From the biochemical point of view, the spaghetti enriched with 10% OPF and 0.6% TG considerably improved the bioactive components of sample. In particular, the PUFA/SFA ratio resulted higher (1.16) than the control (0.69) and the total polyphenols content in dry spaghetti increased from 82.39 μg/g DW to 245.08 μg/g DW. In addition, it was interesting to underline that the spaghetti preparation process preserved an aliquot of flavonoids that are known to be important for their several biological effects. In particular, the results showed that levels of apigenin, luteolin and quercetin, observed in OPF spaghetti, was 15 times higher than the control. In conclusion, the proper addition of OPF and TG for pasta enrichment could represent a means to valorize olive oil industrial by-products.

## Figures and Tables

**Table 1:** Tocochromanols, carotenoids, fatty acids and phenolic composition of olive paste flour (OPF) and semolina Dry Weight (DW), ND: not detectable.

		Olive Paste Flour			Semolina	
Tocochromanols (µg/g DW)						
β T3		ND			11.95 ± 0.71	
α T		107.17 ± 1.78			ND	
Carotenoids (µg/g DW)						
Lutein		4.03 ± 0.27			5.59 ± 0.26	
Zeaxanthin		0.19 ± 0.001			0.25 ± 0.004	
α-carotene		0.45 ± 0.03			ND	
β-carotene		0.69 ± 0.06			0.037 ± 0.001	
Triterpenic acids (mg/g DW)						
Maslinic		6.76 ± 0.45			ND	
Eonolic		3.65 ± 0.32			ND	
Fatty acids (%)						
Myristic C14:0		ND			0.24 ± 0.01	
Palmitic C16:0		21.40 ± 2.70			43.93 ± 0.63	
Palmitoleic C16:1		1.81 ± 0.21			5.96 ± 0.24	
Stearic C18:0		3.25 ± 0.47			5.04 ± 0.15	
Cis Oleic C18:1		57.20 ± 0.24			7.01 ± 0.16	
Trans oleic C18:1		3.08 ± 0.37			0.82 ± 0.02	
Linoleic C18:2		12.50 ± 1.36			35.20 ± 0.50	
Linolenic C18:3		0.79 ± 0.06			1.80 ± 0.04	
Phenols (µg/g DW)						
	Free	Non-Extractable	Total	Free	Conjugated + Bound	Total
Caffeic acid	52.01 ± 2.54	576.70 ± 2.83	628.71 ± 5.37	ND	ND	ND
4-Hydroxybenzoic acid	ND	ND	ND	ND	0.94 ± 0.17	0.90 ± 0.20
Vanillic acid	156.13 ± 7.83	ND	156.13 ± 7.83	0.64 ± 0.07	0.60 ± 0.18	1.20 ± 0.30
Syringic acid	ND	ND	ND	ND	0.71 ± 0.24	0.70 ± 0.20
Cumaric acid	117.74 ± 7.09	39.57 ± 2.33	157.31 ± 9.42	0.25 ± 0.01	1.80 ± 0.34	2.00 ± 0.35
Ferulic acid	ND	16.46 ± 2.31	16.46 ± 2.31	0.67 ± 0.04	72.50 ± 8.54	73.17 ± 8.78
Sinapic acid	ND	ND	ND	0.21 ± 0.01	8.50 ± 1.30	8.71 ± 1.31
Caffeic acid derivative	ND	ND	ND	ND	1.41 ± 0.34	1.41 ± 0.30
Apigenin derivate	ND	ND	ND	0.74 ± 0.38	ND	0.74 ± 0.38
Vitexin	ND	ND	ND	2.36 ± 1.21	ND	2.36 ± 1.21
Quercetin 3-*O*-glucoside	72.90 ± 3.81	ND	72.90 ± 3.81	ND	ND	ND
Glicosylated luteolin derivate	27.31 ± 0.91	ND	27.13 ± 0.91	ND	ND	ND
Quercetin derivate	308.23 ± 34.36	ND	308.23 ± 34.36	ND	ND	ND
Luteolin	532.59 ± 53.91	ND	532.59 ± 53.91	1.07 ± 0.08	ND	1.07 ± 0.08
Apigenin	30.06 ± 3.02	ND	30.06 ± 3.02	0.10 ± 0.02	ND	0.10 ± 0.02
Luteolin derivate	12.24 ± 1.24	ND	12.24 ± 1.24	ND	ND	ND
Tyrosol	932.12 ± 42.13	ND	936.12 ± 42.13	ND	ND	ND
Oleuropein	371.42 ± 25.41	ND	371.42 ± 25.41	ND	ND	ND
Total	2616.57 ± 182.25	632.73 ± 7.47	3249.30 ± 189.72	6.04 ± 1.84	86.50 ± 11.30	92.54 ± 13.10

**Table 2:** Sensory characteristics of uncooked and cooked dry spaghetti samples investigated in the Step 2.

	Uncooked Spaghetti			Cooked Spaghetti								
	Color	Resistance to Break	Overall Quality	Elasticity	Firmness	Fibrous	Bulkiness	Adhesiveness	Color	Odor	Taste	Overall Quality
CTRL	7.20 ± 0.27 ^a^	7.27 ± 0.28 ^a^	7.23 ± 0.26 ^a^	7.08 ± 0.28 ^a^	7.21 ± 0.35 ^a^	7.02 ± 0.31 ^a^	6.14 ± 0.33 ^a^	6.21 ± 0.26 ^a^	7.56 ± 0.38 ^a^	7.23 ± 0.28 ^a^	7.01 ± 0.34 ^a^	7.20 ± 0.26 ^a^
10% OPF	6.07 ± 0.30 ^b^	6.20 ± 0.27 ^b^	6.20 ± 0.27 ^b^	5.20 ± 0.26 ^b^	6.24 ± 0.30 ^b^	5.80 ± 0.25 ^b^	5.27 ± 0.30 ^b^	5.31 ± 0.34 ^b^	6.72 ± 0.32 ^b^	6.98 ± 0.30 ^a^	6.71 ± 0.36 ^a^	5.32 ± 0.32 ^b^
15% OPF	5.43 ± 0.37 ^b^	5.20 ± 0.27 ^c^	5.34 ± 0.38 ^c^	4.20 ± 0.26 ^c^	5.80 ± 0.30 ^b^	4.80 ± 0.25 ^c^	4.77 ± 0.41 ^b^	4.95 ± 0.28 ^b^	5.36 ± 0.50 ^c^	6.18 ± 0.30 ^b^	5.84 ± 0.27 ^b^	4.42 ± 0.36 ^c^

^a–c^ Mean in the same column followed by different superscript letters differ significantly (*p* < 0.05). OPF: Olive Paste Flour.

**Table 3:** Sensory characteristics of uncooked and cooked dry spaghetti samples investigated in the Step 3.

	Uncooked Spaghetti			Cooked Spaghetti								
	Color	Resistance to Break	Overall Quality	Elasticity	Firmness	Fibrous	Bulkiness	Adhesiveness	Color	Odor	Taste	Overall Quality
CTRL	7.20 ± 0.27 ^a^	7.27 ± 0.28 ^a^	7.23 ± 0.26 ^a^	7.08 ± 0.28 ^a^	7.21 ± 0.35 ^a^	7.02 ± 0.31 ^a^	6.20 ± 0.28 ^a^	6.05 ± 0.26 ^a^	7.56 ± 0.38 ^a^	7.23 ± 0.28 ^a^	7.01 ± 0.34 ^a^	7.20 ± 0.26 ^a^
10% OPF	6.07 ± 0.30 ^b^	6.20 ± 0.27 ^c^	6.20 ± 0.27 ^b^	5.20 ± 0.26 ^b^	6.24 ± 0.30 ^b^	5.80 ± 0.25 ^b^	5.27 ± 0.30 ^b^	5.31 ± 0.34 ^b^	6.72 ± 0.32 ^b^	6.98 ± 0.30 ^a^	6.71 ± 0.36 ^a^	5.32 ± 0.32 ^b^
10% OPF-0.3 TG	6.00 ± 0.30 ^b^	6.41 ± 0.36 ^b,c^	6.55 ± 0.36 ^b^	5.30 ± 0.35 ^b^	6.26 ± 0.31 ^b^	5.82 ± 0.20 ^b^	5.61 ± 0.38 ^b^	5.41 ± 0.38 ^b^	6.75 ± 0.28 ^b^	7.00 ± 0.31 ^a^	6.73 ± 0.36 ^a^	5.73 ± 0.36 ^b^
10% OPF-0.6 TG	6.05 ± 0.26 ^b^	6.85 ± 0.30 ^a,b^	6.77 ± 0.31 ^a,b^	5.65 ± 0.21 ^b^	6.31 ± 0.30 ^b^	5.86 ± 0.21 ^b^	6.55 ± 0.25 ^a^	6.08 ± 0.28 ^a^	6.21 ± 0.33 ^b^	7.03 ± 0.32 ^a^	6.83 ± 0.25 ^a^	6.56 ± 0.33 ^a^

^a,b^ Mean in the same column followed by different superscript letters differ significantly (*p* < 0.05). TG: Transglutaminase.

**Table 4:** Cooking quality of dry spaghetti studied in the Step 3.

	OCT (min)	Cooking Loss (%)	Swelling Index (g Water Per g Dry Spaghetti)	Water Absorption (%)	Adhesiveness (Nmm)	Hardness (N)
CTRL	10.30	5.05 ± 0.28 ^c^	1.86 ± 0.07 ^a^	141 ± 3.64 ^a^	0.69 ± 0.06 ^a^	6.69 ± 0.32 ^c^
10% OPF	9.00	6.20 ± 0.12 ^a^	1.75 ± 0.05 ^b^	138 ± 8.78 ^a,b^	0.78 ± 0.13 ^a^	7.96 ± 0.23 ^b^
10% OPF-0.3 TG	9.30	5.93 ± 0.23 ^a,b^	1.62 ± 0.04 ^c^	128 ± 4.38 ^b,c^	0.68 ± 0.12 ^a^	8.15 ± 0.42 ^b^
10% OPF-0.6 TG	10.00	5.65 ± 0.14 ^b^	1.61 ± 0.02 ^c^	126 ± 2.36 ^c^	0.62 ± 0.13 ^a^	9.55 ± 0.71 ^a^

^a–c^ Mean in the same column followed by different superscript letters differ significantly (*p* < 0.05). OCT: Optimal Cooking Time.

**Table 5:** Tocochromanols, carotenoids, fatty acids and phenolic composition of control spaghetti (CTRL) and spaghetti enriched with 10% olive paste flour (OPF). Different letters indicate differences between the CTRL and OPF (*p* < 0.05). ND: not detectable.

		CTRL			Spaghetti 10% OPF	
Tocochromanols (µg/g DW)						
α T		ND			8.63 ± 0.04	
Carotenoids (µg/g DW)						
Lutein		4.22 ± 0.95 ^a^			4.72 ± 0.09 ^a^	
Zeaxanthin		0.18 ± 0.002 ^a^			0.16 ± 0.02 ^a^	
α-carotene		ND			0.08 ± 0.001	
β-carotene		ND			0.27 ± 0.003	
Total		4.40 ± 0.95 ^a^			5.23 ± 0.11 ^b^	
Triterpenic acids (mg/g DW)						
Maslinic		ND			1.32 ± 0.04	
Eonolic		ND			0.54 ± 0.05	
Total		ND			1.86 ± 0.07	
Fatty acids (%)						
Myristic C14:0		0.37 ± 0.01 ^a^			0.12 ± 0.01 ^b^	
Palmitic C16:0		41.73 ± 0.19 ^a^			23.20 ± 0.32 ^b^	
Palmitoleic C16:1		1.95 ± 0.11 ^a^			2.31 ± 0.09 ^b^	
Stearic C18:0		7.50 ± 0.44 ^a^			2.49 ± 0.29 ^b^	
Cis Oleic C18:1		12.60 ± 2.22 ^a^			38.90 ± 2.41 ^b^	
Trans oleico C18:1		1.48 ± 0.15 ^a^			2.96 ± 0.20 ^b^	
Linoleic C18:2		32.61 ± 1.46 ^a^			28.68 ± 2.75 ^a^	
Linolenic C18:3		1.76 ± 0.18 ^a^			1.34 ± 0.64 ^a^	
Phenols (µg/g DW)						
	Free	Conjugated + bound	Total	Free	Conjugated + bound	Total
Caffeic acid	ND	ND	ND	1.32 ± 0.14	2.25 ± 0.31	3.57 ± 0.45
4-Hydroxybenzoic acid	ND	1.28 ± 0.07 ^a^	1.28 ± 0.07 ^a^	ND	2.81 ± 0.16 ^b^	2.81 ± 0.16 ^b^
Vanillic acid	0.56 ± 0.04 ^a^	0.78 ± 0.01 ^a^	1.34 ± 0.05 ^a^	7.28 ± 0.46 ^b^	1.99 ± 0.62 ^b^	9.27 ± 1.08 ^b^
Syringic acid	ND	0.75 ± 0.08	0.75 ± 0.08	ND	ND	ND
Cumaric acid	0.17 ± 0.01	1.75 ± 0.01 ^a^	1.92 ± 0.02 ^a^	1.22 ± 0.13 ^b^	23.47 ± 1.90 ^b^	24.69 ± 2.03 ^b^
Ferulic acid	0.35 ± 0.02	67.70 ± 0.19 ^a^	68.05 ± 0.21 ^a^	ND	60.93 ± 4.28 ^a^	60.93 ± 4.28 ^b^
Sinapic acid	ND	5.78 ± 0.55 ^a^	5.78 ± 0.55 ^a^	ND	9.79 ± 0.73 ^b^	9.79 ± 0.73 ^b^
Caffeic acid derivative	ND	2.05 ± 0.12	2.05 ± 0.12 ^a^	0.84 ± 0.08	ND	0.84 ± 0.08 ^b^
Apigenin derivate	0.27 ± 0.02	ND	0.27 ± 0.02	ND	ND	ND
Vitexin	0.78 ± 0.12	ND	0.78 ± 0.12	ND	ND	ND
Quercetin 3-*O*-glucoside	ND	ND	ND	ND	ND	ND
Quercetin	ND	ND	ND	5.49 ± 0.89	ND	5.49 ± 0.89
Luteolin	0.17 ± 0.08 ^a^	ND	0.17 ± 0.08 ^a^	11.46 ± 2.16 b	ND	11.46 ± 2.16 ^b^
Apigenin	ND	ND	ND	1.18 ± 0.19	ND	1.18 ± 0.19
Tyrosol	ND	ND	ND	66.10 ± 2.47	36.23 ± 4.52	102.33 ± 6.99
Oleuropein	ND	ND	ND	12.72 ± 1.17	ND	12.72 ± 1.17
Total	2.30 ± 0.29 ^a^	80.09 ± 1.03 ^a^	82.39 ± 1.32 ^a^	107.61 ± 7.69 ^b^	137.47 ± 12.52 ^b^	245.08 ± 20.21 ^b^
